# Global Peak in Atmospheric Radiocarbon Provides a Potential Definition for the Onset of the Anthropocene Epoch in 1965

**DOI:** 10.1038/s41598-018-20970-5

**Published:** 2018-02-19

**Authors:** Chris S. M. Turney, Jonathan Palmer, Mark A. Maslin, Alan Hogg, Christopher J. Fogwill, John Southon, Pavla Fenwick, Gerhard Helle, Janet M. Wilmshurst, Matt McGlone, Christopher Bronk Ramsey, Zoë Thomas, Mathew Lipson, Brent Beaven, Richard T. Jones, Oliver Andrews, Quan Hua

**Affiliations:** 1 Present Address: Palaeontology, Geobiology and Earth Archives Research Centre, School of Biological, Earth and Environmental Sciences, University of New South Wales, Sydney, NSW 2052, Australia; 2 Climate Change Research Centre, School of Biological, Earth and Environmental Sciences, University of New South Wales, Sydney, NSW 2052, Australia; 3 ARC Centre of Excellence in Australian Biodiversity and Heritage, School of Biological, Earth and Environmental Sciences, University of New South Wales, Sydney, NSW 2052, Australia; 4 Department of Geography, University College London, Gower Street, London, WC1E 6BT UK; 5 Waikato Radiocarbon Laboratory, University of Waikato, Private Bag, 3105 Hamilton, New Zealand; 6 Department of Earth System Science, University of California, Irvine, CA 92697-3100 USA; 7 Gondwana Tree-Ring Laboratory, P.O. Box 14, Little River, Canterbury, 7546 New Zealand; 8 GFZ German Research Centre for Geosciences, Section 5.2, Telegrafenberg, 14473 Potsdam, Germany; 9 Long Term Ecology Laboratory, Landcare Research, PO Box 69040, Lincoln, 7640 New Zealand; 10 School of Environment, University of Auckland, Private Bag 92019, Auckland, 1142 New Zealand; 11 Research Laboratory for Archaeology and the History of Art, University of Oxford, Dyson Perrins Building, South Parks Road, Oxford, OX1 3QY UK; 12 Conservation House, PO Box 10420, Wellington, 6143 New Zealand; 13 Department of Geography, University of Exeter, Devon, EX4 4RJ UK; 14 Tyndall Centre for Climate Change Research, School of Environmental Sciences, University of East Anglia, Norwich Research Park, Norwich, NR4 7TJ UK; 15 Australian Nuclear Science and Technology Organisation (ANSTO), Locked Bag 2001, Kirrawee DC, NSW 2232 Australia; 16 School of Geography, Geology and the Environment, University of Keele, Keele, Newcastle-under-Lyme, United Kingdom

## Abstract

Anthropogenic activity is now recognised as having profoundly and permanently altered the Earth system, suggesting we have entered a human-dominated geological epoch, the ‘Anthropocene’. To formally define the onset of the Anthropocene, a synchronous global signature within geological-forming materials is required. Here we report a series of precisely-dated tree-ring records from Campbell Island (Southern Ocean) that capture peak atmospheric radiocarbon (^14^C) resulting from Northern Hemisphere-dominated thermonuclear bomb tests during the 1950s and 1960s. The only alien tree on the island, a Sitka spruce (*Picea sitchensis*), allows us to seasonally-resolve Southern Hemisphere atmospheric ^14^C, demonstrating the ‘bomb peak’ in this remote and pristine location occurred in the last-quarter of 1965 (October-December), coincident with the broader changes associated with the post-World War II ‘Great Acceleration’ in industrial capacity and consumption. Our findings provide a precisely-resolved potential Global Stratotype Section and Point (GSSP) or ‘golden spike’, marking the onset of the Anthropocene Epoch.

## Introduction

Since the nineteenth century geologists have considered the recent environmental impacts of humans^1^ but increasing awareness of the scale and magnitude of change has led to suggestions that we may have entered a new geological epoch^1–6^. A major criterion for the definition of this new geological epoch, known as the Anthropocene, is the presence of a global anthropogenic signature^3^ preserved in the geological record^4,6^ that represents or is coeval with permanent changes in the Earth system. Paul Crutzen and Eugene Stoermer informally placed the onset of the Anthropocene in the mid-eighteenth century, arguing that as a result of industrialisation and urbanisation, atmospheric greenhouse gas concentrations (CO_2_ and CH_4_) moved beyond historic long-term values^2,7^, driving global climate changes^8,9^. Since this proposal, other golden spikes have been suggested, though not all can be unambiguously attributed to human activity. For instance, the onset of the Anthropocene has also been proposed to have occurred in the early part of the Holocene as a result of ‘anomalous’ increases in greenhouse gases^10^, due to deforestation and rice planting associated with the spread of farming. Alternatively, the prominent decrease in atmospheric CO_2_ at CE 1610 has been suggested as a marker, potentially occurring as a result of European arrival in the Americas, leading to plague, death, and reforestation^3^. Both these possible markers for the onset of the Anthropocene, however, may have natural causes^11,12^. It has also been suggested that the ‘Great Acceleration’ in global economic activity, consumption and human population that followed the Second World War could define the epoch^13^ but the potential markers for the onset of this activity are highly spatially variable and diachronous, limiting their application as a global horizon for the base of the Anthropocene.

To define an epoch in the geological timescale^14^ requires the formal identification of either a Global Boundary Stratotype Section and Point (GSSP) or if no candidate can be recognised, a Global Standard Stratigraphic Age (GSSA)^3,15^. To be defined as a GSSP, a sequence at a specific location must preserve an accumulation of material over time that contains a single physical expression of change with no evidence of a hiatus^15,16^ (see Methods). An excellent example in this regard is the Cretaceous-Paleogene (K/Pg) boundary at 66 million years ago identified by an anomalous peak in iridium levels within the lowermost 1–3 mm of a rust-coloured marine clay unit exposed at El Kef (Tunisia)^17^, marking a bolide impact and the associated global extinction of non-avian dinosaurs and explosion in mammal populations and species. Another example is the onset of the Quaternary by interglacial Marine Isotope Stage 103 (ref.^18^). Whilst traditionally most GSSPs utilise marine records that have subsequently been lithified and exposed at the surface as a rock outcrop, there is increasing recognition that GSSPs do not necessarily need to comprise a solid aggregate deposit but may be made up of any geologic stratigraphic material. A recent example is the formal definition of the onset of the Holocene as an abrupt North Atlantic warming at 11,650 years before CE 1950 as recorded in a Greenland ice core^19^.

A major challenge for defining the Anthropocene is that few changes in the Earth system are absolutely synchronous^3,4^. A signal that is transported via the atmosphere, however, has the potential to be near-instantaneous around the world. Above-ground thermonuclear weapon testing across the time interval of the 1940s to 1980s – most of which was in the Northern Hemisphere – generated a host of different radionuclides in the atmosphere, including radiocarbon (^14^C), iodine (^129^I), caesium (^137^Cs) and plutonium (^239^Pu/^240^Pu)^20–23^. Previous work has argued that the beginning of the nuclear age and the global distribution of radioactive nuclides provide an effective marker within the broader Great Acceleration in human activity on our planet^3,20^. As a result, the first atomic bomb test in Alamogordo, New Mexico on 16 July CE 1945 (ref.^20^) and the Northern Hemisphere 1964 ‘bomb peak’ in radionuclides^3,4^ have been considered for the onset of the Anthropocene. Direct fallout of radioactive particles (such as Pu) can be influenced by proximity to nuclear weapon test sites and environmental processes, with relatively low concentrations in the Southern Hemisphere at detection limits and/or the local expression of ‘early’ 1950s tests reaching levels comparable to a decade later^24^. In contrast, the so-called ‘bomb’ radiocarbon was primarily injected into the stratosphere^25^, forming ^14^CO_2_ that was transferred down into the troposphere through the late Northern Hemisphere spring exchange of air masses and subsequently mixed through zonal and meridional atmospheric circulation, ensuring global distribution^26^. As a consequence, the atmospheric ^14^C content (or ∆^14^C) approximately doubled in the Northern Hemisphere to form a peak centred on 1964 but encompassing a broader period, CE 1962 to 1967 (Figure S1; ref.^27^). Following the 1963 Partial Test Ban Treaty and later agreements, atmospheric ∆^14^C levels have decreased through air-sea exchange^28^, fixation by the biosphere^29^ and dilution by fossil fuel emissions^26^. In contrast to radionuclide particles, the relatively slow removal of ^14^C from the troposphere^25^ raises the possibility that the preservation of an atmospheric signal in the geological record may be detected synchronously around the world. Although excess ^14^C associated with thermonuclear testing is preserved in marine sequences (corals and sediments)^30^, the transfer is governed by a number of different factors including local rates of exchange, diffusivity and ocean circulation^26,31^, resulting in spatial and temporal differences in the expression of the bomb peak. The longest series of atmospheric ^14^CO_2_ observations commenced in the Southern Hemisphere in CE 1954 at Lower Hutt, Wellington (New Zealand)^32^, recording the global expression of the bomb peak. Tree-rings incorporate carbon directly from the atmosphere at the time of formation, capturing a record of atmospheric ^14^C content, but preservation of the bomb peak signal in Southern Hemisphere trees remains equivocal. Factors include being directly influenced by Northern Hemisphere air masses in low latitudes^33^, missing/false tree-rings^34^, and/or potential offset by seasonal growth and the assimilation of biospheric decayed CO_2_ (refs^35,36^). Whilst Pu has been detected in tree rings, early work demonstrated that the incorporation of this radioisotope can be highly heterogeneous, both spatially and temporally, with uptake sometimes delayed by decades^37^. To precisely identify the timing of the bomb radiocarbon peak in the Southern Hemisphere requires multiple replication of tree-ring series from an exposed (i.e. pure air), mid-latitude location^35^, thereby capturing a global signal with zero age uncertainty.

Here we report a series of ^14^C measurements from annually-resolved tree-ring chronologies and a sub-decadally resolved peat sequence from the New Zealand subantarctic Campbell Island, an uninhabited UNESCO World Heritage site lying in the Southern Ocean at 52°S in the core latitude of Southern Hemisphere westerly airflow^38,39^. For the trees, we exploited large specimens of two native species *Dracophyllum scoparium* and *D. longifolium*^40^, and a seasonally-resolved chronology of a single Sitka spruce (*Picea sitchensis*)^41^, the only alien tree on the island, with an order of magnitude larger growth rings than the *Dracophyllum* and a proven ability to rapidly assimilate atmospheric ^14^CO_2_ (refs^35,42^). We recognise a southern ^14^C maximum in these geological-forming sequences during October-December 1965 – falling within the broad CE 1962–1967 peak observed in the Northern Hemisphere^3,4^ – thereby providing a truly global marker for the onset of the Anthropocene Epoch.

## Results

**Figure 1:**
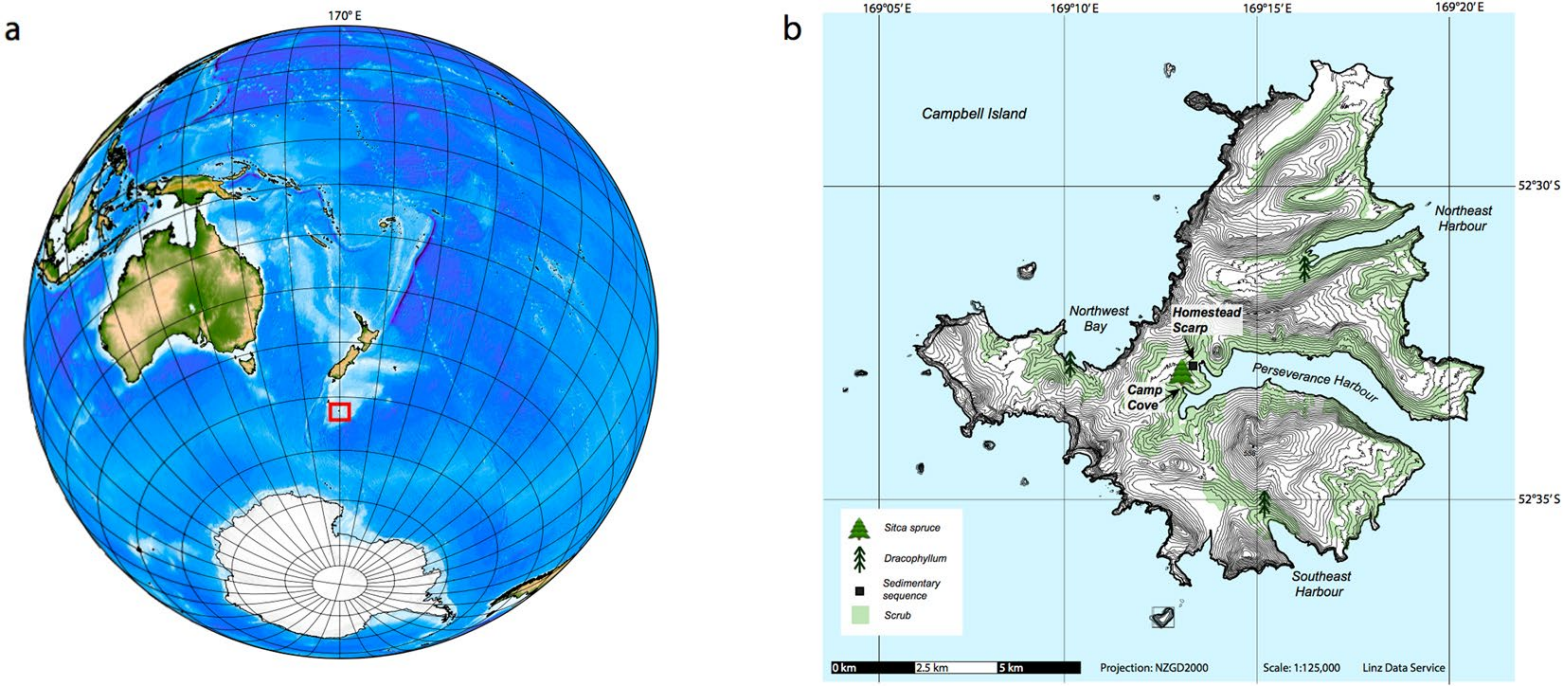
Defining the global onset of the Anthropocene Epoch in the Southern Ocean. Location of New Zealand subantarctic Campbell Island (red box, panel a), sedimentary and tree-ring sites including the Sitka spruce in Camp Cove (panel b). Panels a and b were generated using GMT (Generic Mapping Tools) version 5.2.1 (ref.^71^) and from Land Information New Zealand (LINZ; http://www.linz.govt.nz/) respectively.

**Figure 2:**
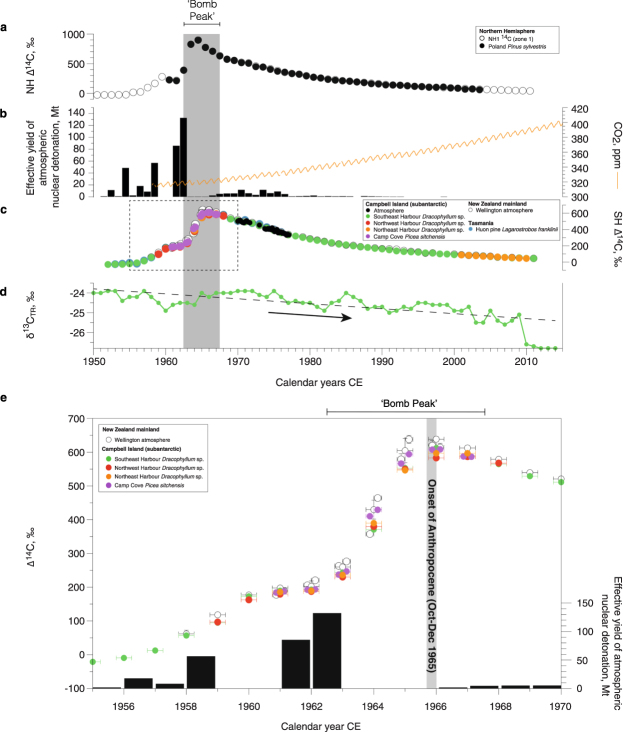
Anthropogenic impacts on the global atmosphere during the twentieth century. Comparison between Northern Hemisphere Zone 1 (NH1) and the Poland pine (*Pinus sylvestris*) atmospheric ^14^C since CE 1950 (refs^51,72^; panel a), effective yield of nuclear bomb detonations^43^ and atmospheric CO_2_ from Mauna Loa (https://www.esrl.noaa.gov/gmd/ccgg/trends/data.html) (panel b), and atmospheric ^14^C as recorded over Lower Hutt (Wellington)^32^ and Campbell Island^44^, plotted against absolutely dated tree-ring sequences from Tasmania^36^ and subantarctic Campbell Island^40,41^ (panel c). The bomb peak period of 1962–1967 (inclusive) is defined by periods of common ∆^14^C values using regime shift analysis^73^ (95% confidence; Figure S1). Stable carbon isotopes in *Dracophyllum* tree SE03 showing twentieth century dilution of atmospheric δ^13^C are presented in panel d; trend shown by dashed line. Panel e shows expanded view of the period 1955–1970 (dashed box in panel c). Only those years in which a minimum of three measurements were made over the six-month austral growing season are plotted for Lower Hutt. Note the peak in the Campbell Island Sitka spruce (*Picea sitchensis*) ^14^C (filled purple circles) during the austral spring (October-December) of 1965, capturing the signal measured at Lower Hutt (open circles) and demonstrating a regionally-representative signal that falls within the period of the Northern Hemisphere bomb peak defined by ref.^27^.

*Dracophyllum* can attain heights over 5 m in sheltered situations^40^, allowing us to generate a well-replicated master chronology back to CE 1870 (see Methods and Supplementary Information). The tree-rings in the *Dracophyllum* were typically ~1 mm in thickness, limiting sampling and ^14^C measurement to annual resolution (see Methods). *Dracophyllum* were sampled at three sites on Campbell Island (Fig. 1). A continuous ^14^C record was obtained from alpha-cellulose extracted from a single specimen at Southeast Harbour (SE15) using Accelerator Mass Spectrometry (AMS). Analysis shows atmospheric radiocarbon over the Southern Ocean first rises during the growing season CE 1954/1955 (October-March), peaking in CE 1965/1966 before gradually declining to the present day (Fig. 2 and Table S4). The greatest ∆^14^C values in the Campbell Island *Dracophyllum* occur some three years after the maximum atmospheric nuclear detonation in the Northern Hemisphere^43^ (Fig. 2). Direct atmospheric observations on Campbell Island^44^, and sub-sampled *Dracophyllum* trees from Northeast Harbour (tree CMB05) and Northwest Harbour (tree NW11) replicate the tree SE15 ^14^C values (Supplementary Information Tables S3 and S4). The signal preserved in the Campbell Island *Dracophyllum* parallels the atmospheric observations made at Lower Hutt^32^ and tree-ring data from Tasmania^36^ indicating the values are representative of the Southern Hemisphere (Fig. 2).

**Figure 3:**
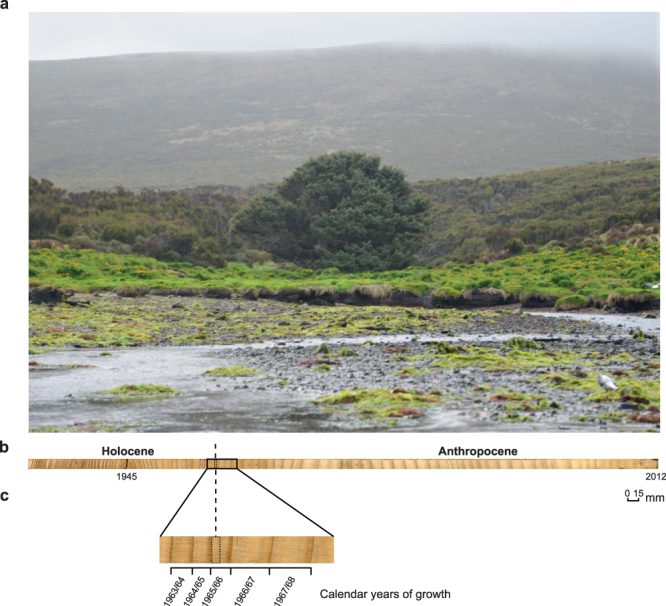
The Loneliest Tree in the World. The subantarctic Campbell Island Sitka spruce (*Picea sitchensis*) surrounded by open *Dracophyllum* sp. scrub (panel a) with visual image of tree-ring growth (panel b) and enlargement of the proposed transition between the Holocene and Anthropocene (panel c).

To refine changing Southern Hemisphere atmospheric ∆^14^C across the bomb peak and minimise the potential for assimilation of biospheric-decayed CO_2_ (ref.^35^), we sampled the Campbell Island Sitka spruce (*Picea sitchensis*), a single example of this alien species on the island, growing in an exposed location in Camp Cove at 52.554°S, 169.133°E (Fig. 3)^41^. Using increment corers we sampled the tree six times, which allowed us to cross-date the samples and indicated no missing rings. The ring thickness was on average an order of magnitude larger than those of the *Dracophyllum*, allowing us to sub-sample the ~1 cm rings during the austral spring (c. October-December) and summer (c. January-March) across the bomb peak. The absolute values of the Sitka spruce ∆^14^C agree with the *Dracophyllum* and the seasonally-derived observations from Lower Hutt (Fig. 2 and Table S4). The Sitka spruce confirms the Southern Hemisphere atmosphere ∆^14^C reached a maximum during the last quarter of 1965 (October-December), falling within the Northern Hemisphere peak of CE 1962–1967 (ref.^27^) (Figure S1).

**Figure 4:**
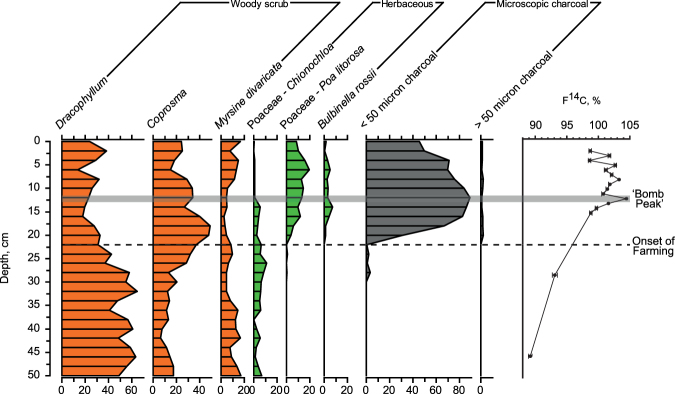
Preservation of the ^14^C bomb peak and evidence of human activity in the Homestead Scarp peat sequence, subantarctic Campbell Island. Summary pollen diagram from Homestead Scarp^38,50^; pollen values calculated as percentage total native terrestrial pollen. Dotted line marks commencement of sheep farming (CE 1931) as indicated by onset of burning and rise of unpalatable species; grey bar denotes the ^14^C ‘bomb peak’.

Whilst the Sitka spruce and the *Dracophyllum* tree-ring sequences may not be regarded traditional contenders for a GSSP, a range of different geologic-forming materials can be considered^3^, as with the Greenland ice core recently utilised to define the onset of the Holocene^19^. To confirm that the bomb peak signal is preserved within a stratigraphic sedimentary record, we undertook contiguous ^14^C measurements through a peat sequence at Homestead Scarp on Campbell Island (Fig. 1; see Methods and Supplementary Information). Elevated ^14^C are observed within the uppermost 14 cm (Table S5), coincident with proxies of human activity on the island since the late 1800s, and a mid-twentieth century peak recorded between 12–13 cm (Fig. 4).

## Discussion

Because CO_2_ produced from fossil fuel emissions is relatively depleted in ^13^C, numerous studies have found a twentieth century decline in atmospheric (and tree-ring) δ^13^C values^45,46^. Although plant δ^13^C can be influenced by climatic and juvenile effects^47,48^, continuous tree-ring series have the potential to provide a proxy of the Great Acceleration. To place the bomb peak in context, we produced a 124-year long record (δ^13^C_TR_) from a *Dracophyllum* tree in Southeast Harbour (Fig. 1; see Methods). The series shows a long-term decline in δ^13^C_TR_ values from −23.2‰ in the late 1920s to −25.5‰ in the early twenty-first century (Figures 2 and S4). There is no discernible shift in δ^13^C_TR_ across the peak in ∆^14^C during late 1965 but the overall depletion in ^13^C demonstrates the bomb peak falls within an environmental signal of accelerating industrialisation and economic growth (i.e., the Great Acceleration)^49^, providing a golden spike for what would otherwise be a series of time-transgressive signals of human activity around the world.

The peak in atmospheric radiocarbon content captured by the trees is also preserved within sedimentary records on Campbell Island, as determined in the Homestead Scarp sequence (Fig. 4). The maximum ^14^C content within the peat sediments at 12–13 cm is coincident with high charcoal and a rise of species unpalatable to sheep (*Bulbinella* spp. and *Poa littorosa*), the latter providing clear evidence of local human activity through farming from CE 1895 (ref.^50^); after 1931, sheep grazing continued with the abandoned stock but were finally removed in 1987. Photographic evidence from the late 1890s and 1907 shows little scrub on the bog surface implying it had already been exposed to fires. It therefore seems likely the small amount of charcoal before the main peak represents early fires associated with sealing activities on the island.

We consider the alien Sitka spruce and associated *Dracophyllum* trees on Campbell Island provide excellent candidates for defining the base of the Anthropocene. Firstly, to be considered a GSSP, the signal should not be modified by natural processes during or after fixation into the ‘geological’ record. Comparison of the *Dracophyllum* (SE15) and Lower Hutt ^14^C records over the bomb peak yields a Pearson correlation of 0.998 (*p* < 0.0001), demonstrating the trees are faithfully mirroring atmospheric content. Our atmospheric ^14^C datasets provide a decade longer record of atmospheric ^14^C than the Polish (Northern Hemisphere) King Castle pine record previously suggested as a GSSP for the Anthropocene (Fig. 2)^3,51^. Here we recognise the same signal in a neobiota on a remote subantarctic island, providing a truly global marker of human impact. Although contemporary atmospheric ^14^C continues to be laid down each year as new tree rings in the Sitka spruce and *Dracophyllum*, the bomb peak is preserved in the inert heartwood, providing a geological stratigraphic material^52^. Secondly, for a signal that was primarily generated in the Northern Hemisphere, its detection in the far south and in a region remote from the impacts of air pollution (in this case localised ^14^C-free fossil fuel CO_2_) confirms the marker as global. Whilst not at the resolution of the tree-ring records, the Homestead Scarp peat sequence provides an auxiliary stratotype that further confirms the ^14^C peak is global. We therefore propose the peak in ∆^14^C during late 1965 within the alien Sitka spruce on Campbell Island to be an excellent GSSP candidate for the base of the Anthropocene (Fig. 3).

We suggest a major advantage of defining the onset of the Anthropocene at the peak in radiocarbon is the associated steep and extreme rise in ^14^C values. Since the half-life of ^14^C is 5730 ± 40 years, the peak provides a long-term boundary, akin to the K/Pg GSSP defined by an iridium peak^17^ and the onset of the Quaternary by interglacial Marine Isotope Stage 103 (ref.^18^). Importantly, the peak will be present at measurable levels in radiocarbon records for tens of thousands of years, allowing its use by many generations of future geologists. The exaggerated nature of the bomb peak is an important consideration for defining the base of the Anthropocene, made more acute by the substantial anticipated dilution of atmospheric concentrations under a range of increasing emissions from fossil fuels (Fig. 2)^53^, and would argue against the use of the onset of bomb tests and initial rise in ∆^14^C from the 1950s (ref.^20^). The atmospheric maximum in radiocarbon in 1965 has been observed to fall on the steep rise in ^14^C within marine sediments and carbonates^30^, providing a global marker across different environments. Furthermore, the association between ^14^C and other radionuclides produced during nuclear bomb tests (e.g., ^129^I and ^239^Pu/^240^Pu) preserved in marine and terrestrial records satisfies the requirement that a signal will be identified in sequences millions of years into the future, far beyond the dating limit of radiocarbon (albeit at lower resolution than the tree ring series reported here)^21,22^.

## Methods

### Tree site locations

We took multiple tree cores from open scrub vegetation to develop cross-dated tree ring chronologies from Campbell Island, a New Zealand subantarctic island and UNESCO World Heritage site in the southwest Pacific Ocean^40,41^. Tree ring samples were taken from the following locations (Fig. 1):

1. Sitka spruce also known as ‘The Loneliest Tree in the World’ and the ‘Christmas Tree’ (*Picea sitchensis*) in Camp Cove (52.554°S, 169.133°E);
2. *Dracophyllum longifolium* from Southeast Harbour (52.59°S, 169.17°E);
3. *Dracophyllum longifolium* from Northeast Harbour (52.52°S, 169.22°E); and
4. *Dracophyllum longifolium* from Northwest Harbour (52.55°S, 169.08°E).

### Tree-ring dating

Thirty *Dracophyllum* spp. trees were sampled, dried then glued on to core mounts and sanded using progressively finer grades of sandpaper to produce a highly-polished surface. The cores were then studied under a binocular microscope and the associated ring patterns cross-dated to ensure a reliable record of growth was captured by the samples taken (i.e. any missing rings/years were identified); a prerequisite for the development of a tree-ring chronology^54^. Every tree-ring was measured to the nearest 0.001 mm using a Velmex measuring stage (www.velmex.com) linked to computer facilities and the measurement series of each core were then standardised to remove biological trends using the dplR program library in R v1.6.4 (ref.^55^). The Sitka spruce and *Dracophyllum* series were combined to produce chronologies for both species (see Supplementary Information Figures S2 and S3, Tables S1 and S2). Within the program, various options are available for the conversion of the annual ring-width measurements into indices and we adopted the use of a more flexible regression model, the Friedman’s Super Smoother^56^, to remove the growth trends. In the case of the single Sitka spruce, normally multiple trees are combined to produce a chronology but the reproducibility of the tree-ring series (and the coherence of the ^14^C signal in the Sitka spruce and *Dracophyllum*; see below) indicates no missing rings in our cores. The annual (*Dracophyllum*) and sub-annual (Sitka spruce) samples were taken by first making fine radial cuts with a band saw for each decade of growth, followed by finer sampling using a clean scalpel.

### Sedimentary sequence

Homestead Scarp is an escarpment situated on the edge of a large domed raised bog (52.55°S, 169.13°E) at 30 m elevation, approximately 440 m inland west of Tucker Cove in Perseverance Harbour, surrounded by 2–3 m high open *Dracophyllum* scrub. The site lies 640 m directly north-northeast of the Sitka spruce tree. The maximum depth of highly organic peat soil at this site is 4 m. The full Lateglacial and Holocene vegetation and climate history derived from pollen data from this site covers the last 17,000 calibrated years^38,50^. A series of pits were dug into the exposed peat face on the edge of the bog, scraped clean, and the profiles collected in large, half-cut drainpipes. Samples were wrapped in polythene wrap in the field and kept cool prior to being stored in a cold store (at 4 °C). Standard methods were used for pollen^57^ and charcoal^58^ analysis. The pollen sum consisted of all pollen and spore types of at least 250 palynomorphs. Microscopic charcoal counts are expressed as a percentage of the pollen sum. Radiocarbon dating of the profile demonstrates that since the mid twentieth century, Homestead Bog has accumulated at the rate of 2.7 cm/decade^−1^ (see below). The site was heavily burned during early farming but since abandonment there have been no fires^50,59^. Given the site’s location on an escarpment and exposure to the pervasive westerly winds, we consider the relatively high charcoal concentration in the upper layers of the Homestead Scarp sequence to be the result of material incorporated from decaying burnt *Dracophyllum* trees following initial burning.

### Radiocarbon measurements

For radiocarbon (^14^C) dating of the wood samples, chemical pretreatment resulted in the purification of alpha-cellulose – as this wood fraction is deemed the most reliable for minimizing potential contamination and providing the most robust ^14^C ages required for such high-precision study^60^. An initial solvent extraction (multiple extractions using acetone) was undertaken to remove mobile components (such as resin oils) thereby reducing the potential for bomb ^14^C translocating across the ring boundaries^11^. The alpha-cellulose extraction process involves an initial acid-base-acid pretreatment at 80 °C, with samples treated with 1 N HCl for 60 min, followed by successive 30-min treatments with 1 N NaOH until the supernatant liquid remained clear and then a final 60-min 1 N HCl wash. Holocellulose was then extracted by using successive 30-min treatments of acidified NaClO_2_ at 70 °C until the wood shavings were bleached to a pale-yellow colour. Alpha-cellulose was then prepared by a final treatment with NaOH followed by a further acid wash (1N HCl at 70 °C for 30 min) and repeated washing with distilled water until a pH of >6 was achieved. For the well-humified ombrothropic peat at Homestead Scarp, contiguous 1-cm samples were taken from the surface down to 16 cm, with the peat surface scraped clean before sampling. Sterile, single-edge razor blades were inserted into either side of the sampling depth for each sample to avoid contamination from either side. The peat was highly humified with no obvious changes in the peat stratigraphy. Any obvious woody rootlets and charcoal fragments were removed. The bulk samples were pretreated using an acid-base-acid (ABA) protocol (with multiple base extractions) and then combusted.

All radiocarbon samples were pretreated and graphitised in the Waikato Radiocarbon Laboratory and measured for radiocarbon by accelerator mass spectrometry (AMS) at the University of California at Irvine (UCI). A major advantage of all samples being prepared in one laboratory (Waikato) and measured for ^14^C in a single facility (UCI) is the potential for inter-laboratory inconsistencies to be avoided, reducing the uncertainties in the ^14^C measurements. The full sequence of tree ^14^C measurements were undertaken from 1950 to 2012 using *Dracophyllum* sampled from Southeast Harbour and their ^14^C content was reported as decay-corrected Δ^14^C. To demonstrate the ^14^C signal is representative of atmospheric levels across Campbell Island, contiguous years in selected decades were measured from cross-dated *Dracophyllum* cores taken from Northeast Harbour and Northwest Harbour. To resolve the seasonal signal, sub-annual samples were taken from the Sitka spruce in Camp Cove across the bomb peak (Fig. 2 and Supplementary Information Table S3). The longest global record of direct atmospheric ^14^CO_2_ observations commenced in 1954 at Lower Hutt (Wellington, New Zealand)^32^ and can be obtained from ftp://ftp.niwa.co.nz/tropac/co2/14co2/, providing a direct comparison to the Campbell Island samples (Fig. 2). The ^14^C values across the inferred period of tree growth (October-March) were extracted using the calibration software OxCal 4.2 (ref.^61^). Where fewer than three Lower Hutt atmospheric measurements were made across the inferred period of tree growth, the calculated value for that ‘year’ was excluded; hence the ‘start’ of the record from 1958 (Fig. 2).

### Stable isotope analysis

To provide a long series of δ^13^C measurements as a measure of fossil fuel dilution of atmospheric CO_2_ through the twentieth century and comparison to the ^14^C datasets reported here, we sampled the tree-rings from *Dracophyllum* SE03 (Figure S4). The stable isotope analysis was undertaken at the German Research Centre for Geosciences GFZ (Postdam, Germany)^62^. Individual tree rings were identified under a binocular microscope (20x), dissected and, if necessary, split into chips smaller than 0.5 mm by using a scalpel. Thereafter, tree-ring cellulose was extracted as described by ref.^63^, homogenized and freeze-dried^64^. Samples were then weighed (140–160 µg) into silver capsules (3.2 × 4 mm, IVA Analysentechnik, Meerbusch, Germany). Dual isotope (δ^13^C_TR_ and δ^18^O_TR_) measurements were performed by subsequently converting tree-ring cellulose samples into CO gas utilizing a TC/EA high temperature pyrolysis device (at 1400 °C) coupled online to a DELTA V Plus isotope ratio mass spectrometer (both Thermo Fisher Scientific, Bremen, Germany). The samples analysed are referenced to standard materials from the International Atomic Energy Agency (IAEA-C3, IAEA-CH6, IAEA-601 and IAEA-602), and checked with secondary standards from Sigma-Aldrich Chemie GmbH, Munich, Germany (Sigma Alpha-Cellulose and Sigma Sucrose) using a two-point normalization method)^65^. Sample replication resulted in a reproducibility of better than ±0.15‰ for δ^13^C values and ±0.25‰ for δ^18^O values. The isotope ratios are given in the δ-notation, relative to the standards V-PDB for δ^13^C and V-SMOW for δ^18^O (ref.^66^). The enrichment in the SE03 *Dracophyllum* ^13^C over the period CE 1889–1928 (Figure S4) most probably represents a juvenile phase of growth which may be the result of shaded growth, incorporation of depleted CO_2_ from the ‘forest’ floor and/or more importantly, changes in tree height and light availability^67^, as well as hydraulic conductivity^48^. Given the open nature of the scrub, the pervasive westerly wind flow over Campbell Island, the absence of a step-change in climate-sensitive δ^18^O_TR_ across the late 1920s, and the statistically significant correlation between the *Dracophyllum* and Lower Hutt ^14^C measurements, we consider incorporation of depleted CO_2_ to be at most only a minor contributor to the radiocarbon signal. In contrast to recent evidence from northern boreal trees^6^, we do not observe any prominent excursion in either stable isotope series that might represent a global signal.

### Definition of a Global Stratotype Section and Type

Formally, a GSSP must satisfy a number of different elements including: a principal correlation event (the marker); other primary and secondary markers; a demonstrated regional and global correlation; complete continuous sedimentation with adequate thickness above and below the marker; an exact location, latitude, longitude and height/depth, as a GSSP is placed at only one place on Earth; be accessible; and have provisions for protection and conservation^3^.

### The Sitka spruce in Campbell Island

The individual *Picea sitchensis* in Camp Cove is also known as ‘the Loneliest Tree in the World’ and located at 52.55°S, 169.13°E (c. 3 metres above sea level)^41^. Planted in open scrub sometime prior to the 1940s and possibly as early as 1903, the tree is now approximately 10 m tall and has a wide, spreading crown consisting of multiple branches, several of which start from near ground level. In this study, two cores from a large lateral branch were taken in 2013 during the Australasian Antarctic Expedition 2013–2014 (www.spiritofmawson.com), and a further four cores were taken during fieldwork in late 2014, demonstrating continuous growth from at least 1941 up to present day with no evidence of missing rings or a slowdown in growth (see Supplementary Information Figure S2); the extent of the branching prevented us from reaching the centre. There have been no reports of cones on the tree during our sampling or previous visits^41,68,69^, implying the tree remains in a prolonged juvenile (pre-reproductive) phase. The present persistently-high levels of rainfall and humidity throughout the year (1376 ± 142 mm/yr; 1941–2015; source: New Zealand National Climate Database, http://cliflo.niwa.co.nz/) are projected to continue into the future under a range of emission scenarios^70^, suggesting the tree poses no threat to the local ecosystem or biodiversity^41^. As a result there are no plans by the New Zealand Department of Conservation to remove the tree from Campbell Island.

The onset of the Anthropocene is defined in the Campbell Island Sitka spruce at Camp Cove by the peak in atmospheric ^14^C preserved in the heartwood of the tree dating to the last quarter of 1965 (within the first half of the tree-ring associated with growth across the 1965/1966 season) (Fig. 2). The proposed GSSP for the base of the Anthropocene is in a remote location, and to examine the Sitka spruce in the field, permission must be obtained from the New Zealand Department of Conservation. However, a simpler solution would be to study the existing cores which are archived by the University of New South Wales (Australia) and the Southland Museum and Art Gallery (New Zealand; http://www.southlandmuseum.co.nz) who can provide free access on request.

### Data availability

All new data is provided in Supplementary Information and will be lodged with the NOAA/World Data Center for Paleoclimatology at https://www.ncdc.noaa.gov/.

## Electronic supplementary material

Supplementary Information
