# Supplementary material for: Global Peak in Atmospheric Radiocarbon Provides a Potential Definition for the Onset of the Anthropocene Epoch in 1965
Source: Sci Rep. 2018 Feb 19;8:3293. doi: 10.1038/s41598-018-20970-5 (PMC5818508; doi:10.1038/s41598-018-20970-5)
Supplement: Supplementary file 1 — Supplementary Information [file 41598_2018_20970_MOESM1_ESM.docx]

**SUPPLEMENTARY INFORMATION**

**Global peak in atmospheric RADIOCARBON provides a Potential definition for THE onset of THE anthropocene EPOCH in 1965**

**Authors**

Chris S.M. Turney^1,2,3*^, Jonathan Palmer^1,2,3^, Mark Maslin^4^, Alan Hogg^5^, Christopher J. Fogwill^1,2,#^, John Southon^6^, Pavla Fenwick^7^, Gerd Helle^8^, Janet M. Wilmshurst^9,10^, Matt McGlone^9^, Christopher Bronk-Ramsey^11^, Zoë Thomas^1,2,3^, Mathew Lipson^2^, Brent Beaven^12^, Richard T. Jones^13^, Oliver Andrews^14^, and Quan Hua^15^.

**Affiliations**

1. Palaeontology, Geobiology and Earth Archives Research Centre, School of Biological, Earth and Environmental Sciences, University of New South Wales, Australia

2. Climate Change Research Centre, School of Biological, Earth and Environmental Sciences, University of New South Wales, Australia

3. ARC Centre of Excellence in Australian Biodiversity and Heritage, School of Biological, Earth and Environmental Sciences, University of New South Wales, Australia

4. Department of Geography, University College London, Gower Street, London, WC1E 6BT, UK

5. Waikato Radiocarbon Laboratory, University of Waikato, Private Bag 3105, Hamilton, New Zealand

6. Department of Earth System Science, University of California, Irvine, CA 92697-3100, USA

7. Gondwana Tree-Ring Laboratory, P.O. Box 14, Little River, Canterbury 7546, New Zealand

8. GFZ German Research Centre for Geosciences, Section 5.2, Telegrafenberg, 14473 Potsdam, Germany

9. Long Term Ecology Laboratory, Landcare Research, PO Box 69040, Lincoln 7640, New Zealand

10. School of Environment, University of Auckland, Private Bag 92019, Auckland 1142, New Zealand

11. Research Laboratory for Archaeology and the History of Art, University of Oxford, Dyson Perrins Building, South Parks Road, Oxford OX1 3QY, UK

12. Conservation House, PO Box 10420, Wellington 6143, New Zealand

13. Department of Geography, University of Exeter, Devon, EX4 4RJ, UK

14. Tyndall Centre for Climate Change Research, School of Environmental Sciences, University of East Anglia, Norwich Research Park, Norwich, NR4 7TJ, UK

15. Australian Nuclear Science and Technology Organisation (ANSTO), Locked Bag 2001, Kirrawee DC, NSW 2232, Australia

#. Current address: School of Geography, Geology and the Environment, University of Keele, United Kingdom

*Corresponding author. e-mail: [c.turney@unsw.edu.au](mailto:c.turney@unsw.edu.au)

**Figure S1:** **Northern Hemisphere (NH1) atmospheric ^14^C content (∆^14^C)^1^**. Horizontal lines denote periods of common ∆^14^C values using regime shift analysis (cut-off length 3 years; 95% confidence)^2^. Grey column defines the ‘bomb peak’ spanning CE 1962 to 1967 inclusive.

**Figure S2:** **Campbell Island tree-ring chronology using Sitka spruce**. Standardised tree-ring indices from the Campbell Island Sitka spruce (*Picea sitchensis*).

**Figure S3:** **Campbell Island tree-ring chronology using *Dracophyllum* spp**. Standardised tree-ring indices of the thirty *Dracophyllum* spp. sampled across Campbell Island. ‘SE’ denotes Southeast Harbour, ‘NW’ Northwest Harbour and ‘CB’ from Northeast Harbour.

**Figure S4:** **Stable carbon isotopic series from Campbell Island *Dracophyllum* spp. over the past century.** δ^13^C and δ^18^O from Southeast Harbour tree 3 (SE03). Reproducibility: 0.12‰ for δ^13^C and 0.27‰ for δ^18^O. Juvenile phase of growth dashed line. Influence of dilution of δ^13^C of atmospheric CO_2_ (the ‘Suess effect’) on tree-ring δ^13^C indicated by plotting twentieth century data of ^13^C of atmospheric CO_2_ extracted from a high-resolution ice core, firn and direct measurements at Cape Grim, Tasmania (blue dots)^3^, as well as long records of direct measurements on flask samples from Baring Head, New Zealand (green line) and Mauna Loa, Hawaii (yellow line)^4^.

**Table S1: Summary descriptive statistics for the six measured tree-ring series from the Sitka spruce at Campbell Island.** The information was compiled using the R software package dplR^5^. ‘AR1’ provides the first order autocorrelation coefficient. The Gini coefficient of inequality which is used as an all-lag measure of diversity in tree-ring records (lower values indicate lower diversity)^6^. ‘Rho’ describes the correlation between a detrended series and a master chronology of all the detrended series. Measurements currently being lodged with the International Tree-Ring Data Bank (ITRDB) at the World Data Center for Paleoclimatology and NOAA National Centers for Environmental Information (NCEI) ([www.ncdc.noaa.gov/data-access/paleoclimatology-data/datasets/tree-ring](http://www.ncdc.noaa.gov/data-access/paleoclimatology-data/datasets/tree-ring)).

**Table S2: Summary descriptive statistics for the thirty measured *Dracophyllum* spp.**

**tree-ring series on Campbell Island.** The information was compiled using the R software package dplR (ref. ^5^). Measurements can be obtained from the International Tree-Ring Data Bank (ITRDB) at the World Data Center for Paleoclimatology and NOAA National Centers for Environmental Information (NCEI) ([www.ncdc.noaa.gov/data-access/paleoclimatology-data/datasets/tree-ring](http://www.ncdc.noaa.gov/data-access/paleoclimatology-data/datasets/tree-ring)).

| **Site series and year** | **Lab code (Wk-)** | **∆^14^C, ‰** | **1σ** |
| --- | --- | --- | --- |
| **Campbell Island**  ***Dracophyllum* SE15, Southeast Hbr** |  |  |  |
| 1952 | 41932 | -29.5 | 1.9 |
| 1953 | 41933 | -27.3 | 2.0 |
| 1954 | 41934 | -27.5 | 1.9 |
| 1955 | 41935 | -21.0 | 1.8 |
| 1956 | 41936 | -9.4 | 1.9 |
| 1957 | 41937 | 12.6 | 1.9 |
| 1958 | 41938 | 57.0 | 2.0 |
| 1959 | 41939 | 94.9 | 2.3 |
| 1960 | 41940 | 172.9 | 2.2 |
| 1961 | 41941 | 189.5 | 2.5 |
| 1962 | 44870 | 192.9 | 2.1 |
| 1963 | 44871 | 243.5 | 2.3 |
| 1964 | 44872 | 370.8 | 2.3 |
| 1965 | 44873 | 552.9 | 2.4 |
| 1966 | 41946 | 612.0 | 3.0 |
| 1967 | 41947 | 589.4 | 3.0 |
| 1968 | 41948 | 564.6 | 3.0 |
| 1969 | 41949 | 528.9 | 2.9 |
| 1970 | 41950 | 511.2 | 2.9 |
| 1971 | 41951 | 491.0 | 2.8 |
| 1972 | 41952 | 478.4 | 2.8 |
| 1973 | 41953 | 449.2 | 2.8 |
| 1974 | 41954 | 415.7 | 2.3 |
| 1975 | 41955 | 388.1 | 2.6 |
| 1976 | 41956 | 360.0 | 2.3 |
| 1977 | 41957 | 333.0 | 2.2 |
| 1978 | 41958 | 320.0 | 2.0 |
| 1979 | 41959 | 302.1 | 2.0 |
| 1980 | 41960 | 281.7 | 2.0 |
| 1981 | 41961 | 266.5 | 2.1 |
| 1982 | 41962 | 250.8 | 2.0 |
| 1983 | 41963 | 238.7 | 1.9 |
| 1984 | 41964 | 219.6 | 1.9 |
| 1985 | 41965 | 212.2 | 2.0 |
| 1986 | 41966 | 198.3 | 2.1 |
| 1987 | 41967 | 187.3 | 1.8 |
| 1988 | 41968 | 179.5 | 1.8 |
| 1989 | 41969 | 168.0 | 2.2 |
| 1990 | 41970 | 160.7 | 1.8 |
| 1991 | 41971 | 150.0 | 1.8 |
| 1992 | 41972 | 136.9 | 1.8 |
| 1993 | 41973 | 129.3 | 1.7 |
| 1994 | 41974 | 124.8 | 1.8 |
| 1995 | 41975 | 120.2 | 1.8 |
| 1996 | 41976 | 112.9 | 1.8 |
| 1997 | 41977 | 110.7 | 1.9 |
| 1998 | 41978 | 100.4 | 1.8 |
| 1999 | 41979 | 98.1 | 2.0 |
| 2000 | 41980 | 90.4 | 2.3 |
| 2001 | 41981 | 87.5 | 1.9 |
| 2002 | 41982 | 82.9 | 1.9 |
| 2003 | 41984 | 78.1 | 2.0 |
| 2004 | 41986 | 72.9 | 1.9 |
| 2005 | 41988 | 66.7 | 1.7 |
| 2006 | 41990 | 60.9 | 1.7 |
| 2007 | 41992 | 58.6 | 1.7 |
| 2008 | 41994 | 50.5 | 1.7 |
| 2009 | 41996 | 52.8 | 1.7 |
| 2010 | 41998 | 46.5 | 1.8 |
| 2011 | 42000 | 45.0 | 1.7 |
|  |  |  |  |
| **Campbell Island**  ***Dracophyllum* CMB05, Northeast Hbr** |  |  |  |
| 1961 | 44261 | 185.4 | 3.0 |
| 1962 | 44262 | 189.3 | 3.0 |
| 1963 | 44263 | 236.1 | 3.0 |
| 1964 | 44264 | 389.6 | 3.0 |
| 1965 | 44265 | 546.2 | 4.0 |
| 1966 | 44266/46254 | 596.6 | 2.2 |
| 1967 | 46255 | 596.6 | 2.8 |
| 2001 | 41983 | 87.1 | 1.7 |
| 2002 | 41985 | 82.4 | 1.8 |
| 2003 | 41987 | 77.0 | 1.8 |
| 2004 | 41989 | 70.5 | 1.8 |
| 2005 | 41991 | 69.4 | 2.0 |
| 2006 | 41993 | 63.5 | 1.8 |
| 2007 | 41995 | 56.3 | 1.8 |
| 2008 | 41997 | 54.7 | 1.9 |
| 2009 | 41999 | 47.7 | 1.8 |
| 2010 | 42001 | 44.6 | 1.7 |
|  |  |  |  |
| **Campbell Island**  ***Dracophyllum* NW11, Northwest Hbr** |  |  |  |
| 1959 | 44404 | 96.8 | 2.0 |
| 1960 | 44405 | 162.6 | 3.0 |
| 1961 | 44406 | 179.4 | 2.0 |
| 1962 | 44407 | 187.3 | 2.0 |
| 1963 | 44408 | 230.1 | 2.0 |
| 1964 | 44409 | 379.7 | 3.0 |
| 1965 | 44410 | 549.2 | 3.0 |
| 1966 | 44411 | 582.9 | 3.0 |
| 1967 | 44412 | 586.7 | 3.0 |
| 1968 | 44413 | 567.6 | 3.0 |
|  |  |  |  |
| **Campbell Island**  **Sitka spruce XT015, Camp Cove** |  |  |  |
| 1960 (spring) | 44552 | 184.2 | 1.5 |
| 1961 (summer) | 44553 | 187.8 | 1.6 |
| 1961 (spring) | 44554 | 192.6 | 1.8 |
| 1962 (summer) | 44555 | 193.7 | 1.7 |
| 1962 (spring) | 44556 | 237.3 | 1.7 |
| 1963 (summer) | 44557 | 247.0 | 1.7 |
| 1963 (spring) | 44558 | 410.2 | 1.8 |
| 1964 (summer) | 44559 | 429.2 | 1.9 |
| 1964 (spring) | 44560 | 566.2 | 2.2 |
| 1965 (summer) | 44561 | 593.8 | 2.1 |
| 1965 (spring) | 44562 | 607.5 | 2.2 |
| 1966 (summer) | 44563 | 608.2 | 2.0 |
| 1966 (spring) | 46257 | 587.3 | 2.9 |
| 1967 (summer) | 46256 | 586.3 | 2.6 |

**Table S3: Radiocarbon (∆^14^C) measurements from the Campbell Island Sitka spruce and *Dracophyllum* tree-ring series.** Years centred on calendar period of growth, with ‘spring’ denoting first half of austral growing season (approximately October-December), and ‘summer’ representing second half of the austral growing season (approximately January-March).

| **Tree** | **Year** | **Δ^14^C, ‰** | **1σ** |
| --- | --- | --- | --- |
| Southeast Harbour | 1965 | 552.9 | 2.4 |
|  | **1966** | **612.0** | **3.0** |
|  | 1967 | 589.4 | 3.0 |
|  |  |  |  |
| Northeast Harbour | 1965 | 546.2 | 4.0 |
|  | **1966** | **596.6^** | **2.2^** |
|  | **1967** | **596.6** | **2.8** |
|  |  |  |  |
| Northwest Harbour | 1965 | 549.2 | 3.0 |
|  | **1966** | **582.9** | **3.0** |
|  | **1967** | **586.7** | **3.0** |
|  | 1968 | 567.6 | 3.0 |
|  |  |  |  |
| Sitka spruce | 1964 (Spring) | 566.2 | 2.2 |
|  | 1965 (Summer) | 593.8 | 2.1 |
|  | **1965 (Spring)** | **607.5** | **2.2** |
|  | **1966 (Summer)** | **608.2** | **2.0** |
|  | 1966 (Spring) | 587.3 | 2.9 |
|  | 1967.125 (Summer) | 586.3 | 2.6 |

**Table S4: Local maxima in Δ^14^C for measured trees on Campbell Island.** Rows in bold show statistically significant peaks in Δ^14^C. Years are centred on calendar period of growth (i.e., January of the calendar year centred on the inferred six-month period of growth). Two highlighted rows indicate the values are statistically indistinguishable. **^**denotes weighted average of two samples.

| **Depth, cm** | **Lab code (Wk-)** | **F^14^C, %** | **1σ** |
| --- | --- | --- | --- |
| 0-1* | 46484 | *111.6* | *0.2* |
| 1-2 | 46485 | *103.0* | *0.2* |
| 2-3 | 46486 | 98.7 | 0.2 |
| 3-4 | 46487 | 101.8 | 0.2 |
| 4-5 | 46488 | 98.6 | 0.2 |
| 5-6 | 46489 | 102.7 | 0.2 |
| 6-7 | 46490 | 101.2 | 0.2 |
| 7-8 | 46491 | 102.1 | 0.2 |
| 8-9 | 46492 | 103.3 | 0.2 |
| 9-10 | 46493 | 101.8 | 0.2 |
| 10-11 | 46494 | 101.4 | 0.2 |
| 11-12* | 46495 | 100.8 | 0.2 |
| 12-13* | 46496 | 104.5 | 0.3 |
| 13-14* | 46497 | 101.6 | 0.3 |
| 14-15* | 46498 | 99.7 | 0.2 |
| 15-16* | 46499 | 98.8 | 0.2 |
| 28-29 | 17095 | 93.1 | 0.4 |
| 45-46 | 17685 | 89.3 | 0.3 |

**Table S5: Radiocarbon measurements from the Homestead Scarp peat exposure, subantarctic Campbell Island.** Asterisk denotes samples with observed macrocharcoal fragments. Although charcoal fragments were largely avoided during sample preparation and pretreatment, the enriched ^14^C measurements from the surface most likely represents the presence of ‘older’ charcoal from the bomb peak (italicised F^14^C values), possibly remobilised during the creation of the exposure.

**References**

1 Hua, Q., Barbetti, M. & Rakowski, A. Z. Atmospheric radiocarbon for the period 1950–2010. *Radiocarbon* **55**, 2059-2072 (2013).

2 Rodionov, S. N. A sequential algorithm for testing climate regime shifts. *Geophysical Research Letters* **31**, L09204, doi: 09210.01029/02004GL019448 (2004).

3 Rubino, M. *et al.* A revised 1000 year atmospheric δ^13^C-CO_2_ record from Law Dome and South Pole, Antarctica. *Journal of Geophysical Research* **118**, 1-18 (2013).

4 Keeling, R., Piper, S., Bollenbacher, A. & Walker, S. Scripps CO_2_ program. (Scripps Institution of Oceanography, University of California, La Jolla, California, USA, 2013).

5 Bunn, A. G. A dendrochronology program library in R (dplR). *Dendrochronologia* **26**, 115-124 (2008).

6 Biondi, F. & Qeadan, F. Inequality in paleorecords. *Ecology* **89**, 1056–1067 (2008).
